# Mediastinal Myofibroblastic Inflammatory Tumor Compressing the Airway: An Unusual Cause of Dyspnea in a 12-Year-Old

**DOI:** 10.7759/cureus.34596

**Published:** 2023-02-03

**Authors:** Camila Tautiva, Gloriana Loria, Manuel E Soto

**Affiliations:** 1 Pediatrics, Hospital Nacional de Niños, Dr Carlos Saenz Herrera, San José, CRI; 2 Pediatric Pulmonology, Hospital Nacional de Niños, Dr Carlos Sáenz Herrera, San José, CRI

**Keywords:** tumor, dyspnea, wheezing, inflammatory miofibroblastic tumor, mediastinum

## Abstract

A healthy 12-year-old presented with wheezing and progressive dyspnea over a 10-month period. He had several general physician consultations and emergency visits during this time and was treated as an asthma exacerbation with no clinical response. He was referred to the pediatric pulmonologist and a tracheal deviation in his previous two chest X-rays was noted, therefore further studies were performed. A severe extrinsic tracheal compression due to a mediastinal mass was documented. He was taken into surgery where a partial resection of the tumor was made. The biopsy of the tumor reported an inflammatory myofibroblastic tumor (IMT), a rare tumor with an atypical presentation, which made this case a diagnostic challenge.

## Introduction

Inflammatory myofibroblastic tumors (IMT) are rare and their diagnosis is based on the histopathological finding of inflammatory cells, fibroblasts, and myofibroblasts [1-2]. They usually appear in children and adolescents and have variable locations, the lung being the most frequent one [2-4]. We present the case of a 12-year-old child with sudden onset of wheezing and dyspnea in whom a mediastinal mass with significant airway obstruction was found. Pathology confirmed the diagnosis, and he was successfully managed with a surgical resection.

This article was previously presented as a meeting abstract at the 2021 International Congress on Pediatric Pulmonology "CIPP 2021".

## Case presentation

A previously healthy 12-year-old, with a family history of asthma, presented with a sudden onset of wheezing and progressive dyspnea over a 10-month period. He had several consultations and emergency visits during this time where he was treated with bronchodilators and started on inhaled steroids with no clinical response.

**Figure 1:**
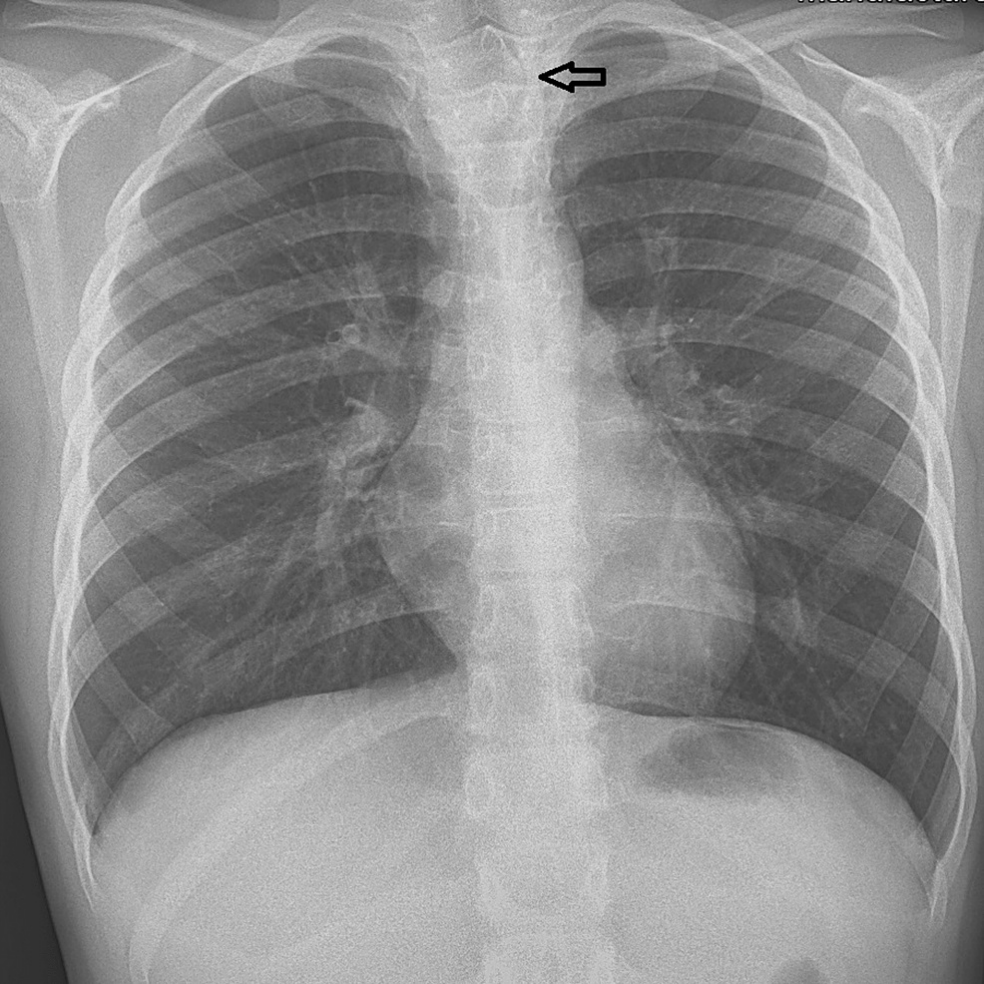
Chest X-ray A clear tracheal deviation can be seen in the chest X-ray.

Due to his persistent symptoms, he was referred to a pediatric pulmonologist where a tracheal deviation in his previous chest X-rays was noted (Figure 1).

**Figure 2:**
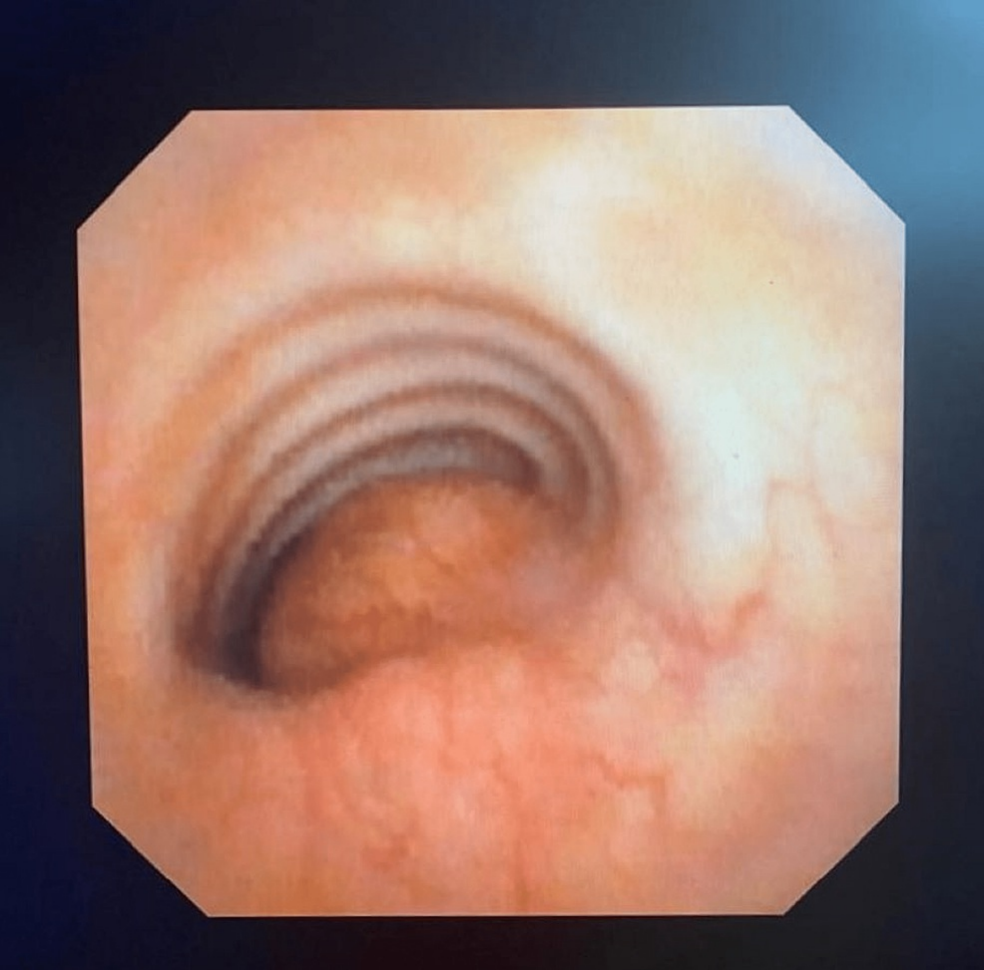
Bronchoscopy Extrinsic tracheal compression occluding 90% of the tracheal lumen

Further studies were performed, and a bronchoscopy documented severe extrinsic tracheal compression due to a mediastinal mass (Figure 2).

**Figure 3:**
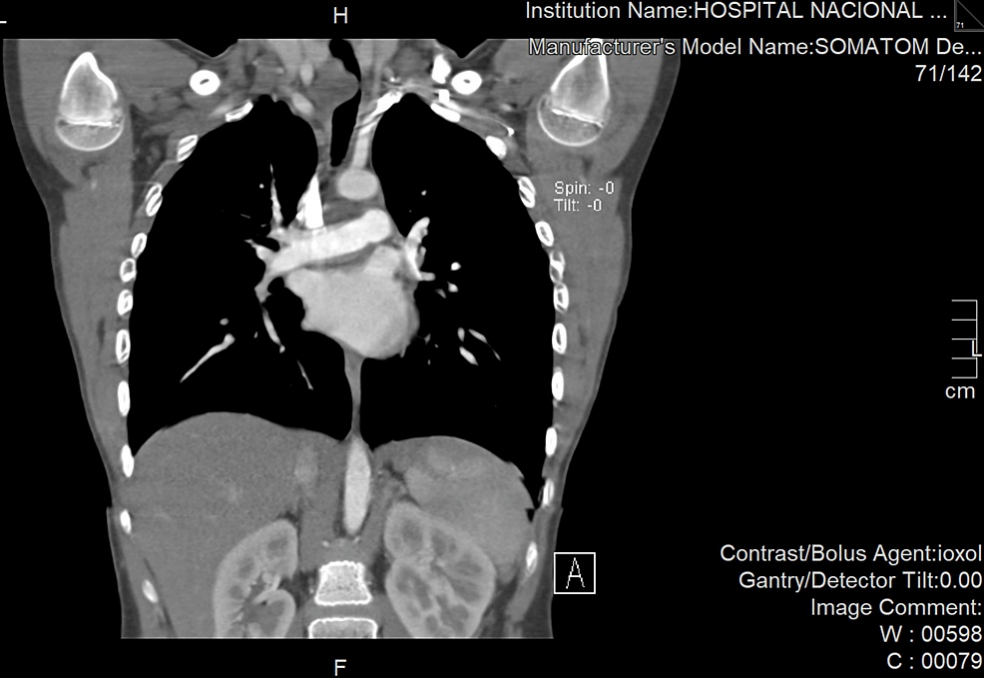
Chest CT A 16x22x30 mm heterogenous mediastinal mass at the right posterolateral level of the trachea

On a chest computerized tomography (CT), a 16x22x30mm heterogenous mediastinal mass was documented at the right posterolateral level of the trachea, which generated an extrinsic compression of 90% of the tracheal lumen (Figure 3).

**Figure 4:**
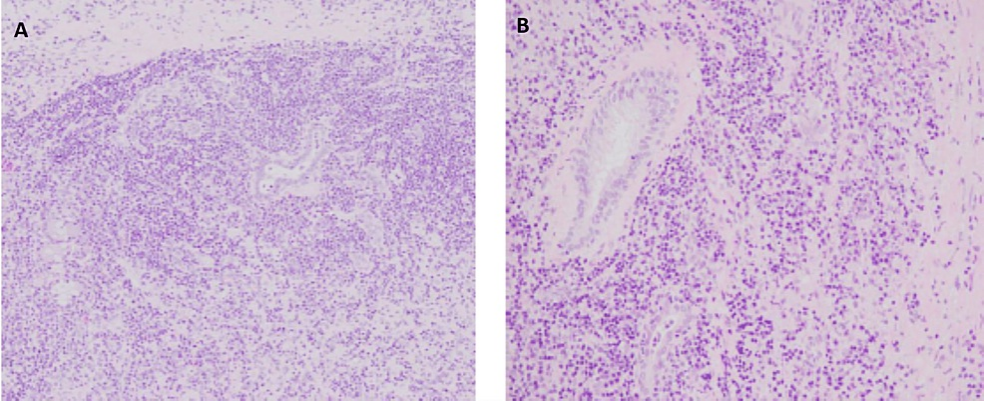
Biopsy results 4A. x100. Myofibroblasts and inflammatory cells are arranged in a scattered manner in a stroma densely fibrocollagenous. 4B. x400. Enlarged oval to spindle-shaped myofibroblasts with clear eosinophilic cytoplasm and oval to spindle-shaped nuclei, arranged diffusely and among which abundant lymphocytes and plasma cells are evident; they tend to arrange in groups without nodular formations. There are also abundant blood vessels, some of which show reactive endothelial hyperplasia. There is a presence of isolated companion cells such as eosinophils, histiocytes, and polymorphonuclear cells.

Multidisciplinary management was offered along with oncology, thoracic surgery, and otorhinolaryngology specialists. Resection of the mass was performed in a two-time surgery, and a tracheostomy was placed to protect airway permeability. Biopsy reported enlarged myofibroblastic cells arranged in a diffuse manner, among which were abundant mature lymphocytes and plasma cells, compatible with an IMT. ALK was negative as well as p53, S-100, CD117, CD34, and ROS-1 (Figure 4).

Our patient had a successful postoperative recovery and was decannulated without complications two months later.

## Discussion

Inflammatory pseudotumors encompass a wide range of neoplastic and non-neoplastic entities, including the inflammatory myofibroblastic tumor (IMT) [1]. These are indolent mesenchymal tumors that usually affect children and young adults and are more prevalent in men, such as our patient [2].

Its etiology is multifactorial and has been associated with trauma, inflammatory conditions, and infections [2,3]. Rearrangement of the ALK gene at locus 2p23 is seen in up to 50% of cases, however, it wasn’t found in our patient. Neither were other genetic markers that could predispose our patient to future neoplasms. Most cases have a benign clinical course, and many are asymptomatic, but up to 15-30% have associated symptoms such as fever, weight loss, fatigue, and symptoms related to their anatomical location [2-4].

IMTs are most often found in the lung, abdomen, and pelvis, but they can also appear in the mediastinum and tracheobronchial three [2,5,6]. Among other possible diagnoses of a mediastinal mass; thymomas, neurogenic tumors, cysts, lymphomas, and germ cell tumors have to be considered [4,7-9]. Mediastinal IMTs are unusual and most published information comes from case reports [7-10]. In a review, they found that in these cases, the most common symptom was dyspnea due to obstruction caused by the tumor, which was the main symptom of our patient [6].

Histologically, it is defined as a lesion composed of tangles of myofibroblastic cells accompanied by an inflammatory infiltrate of plasma cells, lymphocytes, and eosinophils [3,11]. These findings are a requirement for the diagnosis of IMT, and it was with the result of the biopsy that the diagnosis was achieved in our case.

The treatment reported in the literature is mostly surgical resection [2-3]. Our patient successfully underwent surgery and remained asymptomatic afterward.

## Conclusions

Inflammatory myofibroblastic tumors are infrequent and their location at the mediastinum is uncommon. When there is airway compression, the diagnosis of nonspecific symptoms, such as dyspnea, can be challenging for the clinician. This case highlights the importance of an adequate differential diagnosis process and a multidisciplinary approach when a patient presents atypically and does not respond to the usual treatment.
